# Exploring how plasma- and muscle-related parameters affect trout hemolysis as a route to prevent hemoglobin-mediated lipid oxidation of fish muscle

**DOI:** 10.1038/s41598-022-16363-4

**Published:** 2022-08-04

**Authors:** Semhar Ghirmai, Haizhou Wu, Michael Axelsson, Takashi Matsuhira, Hiromi Sakai, Ingrid Undeland

**Affiliations:** 1 Division of Food and Nutrition Science, Department of Biology and Biological Engineering, Chalmers University of Technology, 412 96 Gothenburg, Sweden; 2 Department of Biological and Environmental Sciences, Gothenburg University, Medicinaregatan 18a, 413 90 Gothenburg, Sweden; 3 Department of Chemistry, Nara Medical University, 840 Shijo-cho, Kashihara, Nara 634-8521 Japan

**Keywords:** Lipid peroxides, Membrane lipids, Cell death

## Abstract

Hemoglobin (Hb) is a powerful promoter of lipid oxidation, particularly in muscle of small pelagic fish species and fish by-products, both having high Hb-levels and highly unsaturated lipids. As Hb is located within the red blood cells (RBCs) it is here hypothesized that the perishable polyunsaturated fatty acids (PUFAs) can be protected from oxidation by limiting hemolysis during early fish processing. Using a model system consisting of washed-resuspended trout (*Oncorhynchus mykiss*) RBCs (wr-RBCs), the aim of this study was to evaluate how RBC lysis under cold storage was affected by selected parameters linked to blood or muscle: bacterial growth, energy status, pH, RBC membrane lipid oxidation and colloidal osmotic pressure (COP). The results indicated that bacterial growth had a modest effect on hemolysis while pH-values typical for *post mortem* fish muscle (6.4–6.8), and absence of glucose or albumin stimulated hemolysis. The rapid hemolysis observed at pH 6.4–6.8 correlated with lipid oxidation of the RBC membrane, while the lower hemolysis at pH 7.2–8.0 occurred with low, or without any RBC membrane lipid oxidation. When hemin was added to the RBCs at pH 6.8 hemolysis was induced without parallel RBC membrane oxidation, pointing at Hb-autoxidation and hemin-release per se as important events triggering lysis in fish muscle. Altogether, the study provided valuable findings which ultimately can aid development of new tools to combat lipid oxidation in post mortem fish muscle by limiting hemolysis.

## Introduction

**Figure 1:**
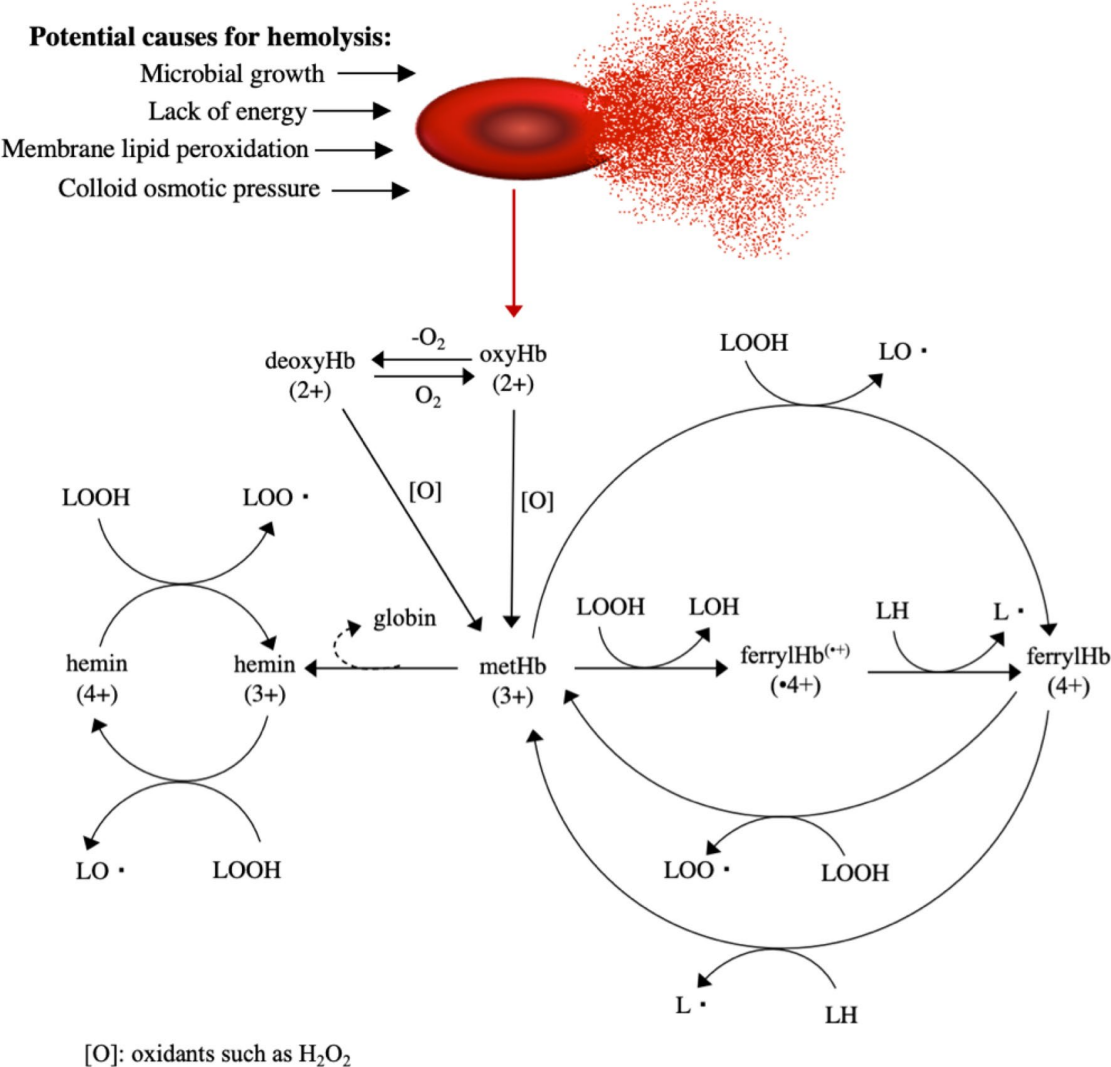
Potential causes for hemolysis and the main pro-oxidative pathways of Hb-mediated lipid oxidation. Modified after Richards^6^.

Lipid oxidation is one of the main causes for post-harvest quality deterioration in muscle based foods and particularly in dark muscle fish such as herring (*Clupea harengus*), sprat (*Sprattus sprattus*) and mackerel (*Scomber scombrus*). These fish are rich in both polyunsaturated fatty acids (PUFA) and heme-proteins; especially hemoglobin (Hb)^1^. Rapid oxidation reduces the nutritional value since the valuable long chain n-3 PUFAs are destroyed and antioxidants such as tocopherol, ubiquinol and ascorbic acid are consumed. Additionally, oxidative reactions can cause changes in texture, color, and development of rancid off-odors and off-flavors^2^. Lipid oxidation in fish muscle is mainly mediated by Hb,a tetrameric protein found inside the red blood cells (RBCs)^3–5^. The two main pro-oxidative pathways for Hb-mediated lipid oxidation in fish (Fig. 1)^6^ are firstly radical formation (LO· and LOO·) via cleavage of pre-formed lipid hydroperoxides (LOOH) by metHb, ferrylHb or hemin. Hemin is more hydrophobic than Hb, thereby more easily interact with the membrane phospholipids^7^. The second pathway follow direct reaction of ferrylHb or ferrylHb radicals with PUFA (LH), generating lipid radicals (L·)^8,9^. The pH-decline naturally taking place in post mortem fish muscle accelerate deoxy- and metHb formation as a result of drastically reduced oxygen affinity due to the Root effect. In the ferric state the porphyrin-group is up to 60-fold less anchored to the globin due to structural changes of the heme crevice^10^, thus hemin-loss is promoted by reduced pH. Further, reduction in pH also generate the formation of hydrogen peroxides (H_2_O_2_) which is the primary deoxy- and metHb oxidant facilitating ferrylHb formation^11^.

Content of residual Hb in fish muscle after bleeding or rinsing is thus of high importance for the fish quality. Bleeding, if performed immediately after capture, before the occurrence of coagulation^12,13^, removes up to ~ 50% of the Hb^3,14^ and has been proven effective in reducing, but not preventing, Hb-mediated lipid oxidation in fish muscle. Bleeding is however not possible with large capture hauls of small pelagic fish species as herring and sprat. For these species, the first point of active blood removal is in the filleting process, in which the fillet is rinsed with tap water. However, as shown in our recent study^15^, the use of tap water is problematic as it causes rapid fish RBC lysis and can thus release Hb into the tissue. Other factors which accelerated RBC lysis in the mentioned study were mechanical stress, elevated temperature up to 12 °C, and hypertonic solutions as simulated sea water. Contrary, whole plasma and certain isolated plasma components as glucose, ascorbic acid (AsA) and albumin prolonged the RBC stability. Despite the importance of Hb removal to stop deterioration of fish muscle quality, and despite earlier findings that limiting hemolysis can delay lipid oxidation in washed fish mince^16^, little is known about causes of hemolysis during post mortem handling, storage and processing of fish. Also, methods to prevent hemolysis during fish processing are, to the best of our knowledge, neither reported on nor commercially implemented.

The aim of this study was to gain better understanding of how selected parameters related to fish muscle and blood affect hemolysis: bacterial growth, pH, energy consumption, RBC membrane lipid oxidation and colloidal osmotic pressure (COP). For this purpose, a model system consisting of washed-resuspended trout RBCs (wr-RBCs) was created and then stored under cold conditions for different amounts of time while following RBC-lysis. Results from this study are expected to contribute to novel strategies for limiting Hb-mediated lipid oxidation at an early stage of the fish process chain. Such knowledge could for instance aid the development of a tailor-made solution for rinsing off blood during fish filleting, or for preservation of fish fillets or blood-rich side-streams such as head and backbone, with minimal hemolysis.

## Materials and methods

### Fish supply

Rainbow trout (*Oncorhynchus mykiss*) was obtained from Vännåns fiskodling AB, Sweden or Samegai Trout Farm, Shiga, Japan. The former fish was maintained in tanks with aerated freshwater, ~ 10 °C, at Gothenburg University, Department of Biological and Environmental Sciences, Zoophysiology. The fish was kept under a 12:12 photoperiod and fed commercially available trout pellets. Results obtained from blood retrieved at the two different fish farms were treated separately. Comparisons of the RBC stability of trout blood from the two locations was performed in plasma or albumin enriched RBC solutions, and similar trends in hemolysis was found in for both locations. Blood from Shiga trout was used in experiments ii and v and blood from Gothenburg trout as used in i, ii, iii and iv.

### Bleeding procedure and preparation of washed red blood cells

The terminal bleeding of trout and the preparation of wr-RBCs was performed as described earlier with minor modification^15^. In short the bleeding procedure comprise the following steps: killing of the fish by a blow to the head and blood was then withdrawln with heparinized syringes from the caudal vessels. Two of the first washing steps of RBCs was performed in 0.9% NaCl with 1 mM Tris–HCl pH 8.0, with the third washing performed in 0.9% NaCl, to remove residual buffer trapped between the RBCs.

The study was performed in compliance with the PREPARE guidelines and the reporting follows the recommendations in the ARRIVE guidelines. All animal procedures were approved by the Gothenburg regional ethical comettee, ethical permit number 5.8.18-06591-2019. At Samegai, Shiga, Japan, trout bleeding was carried out in accordance with the purpose of the Animal Experiment Management Regulations of Nara Medical University.

### Storage of wr-RBCs for studies of hemolysis

**Figure 2:**
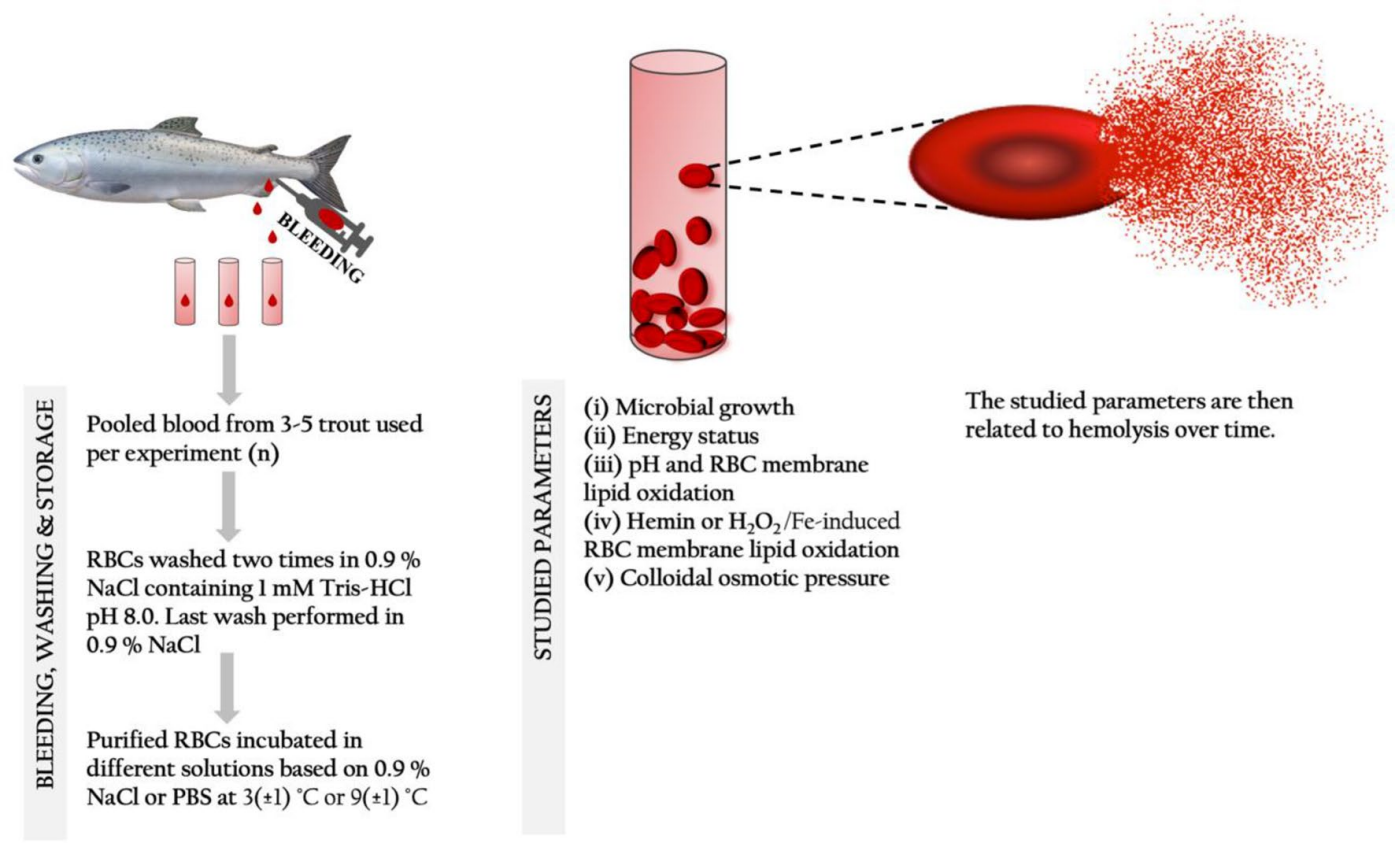
Potential causes for hemolysis and the main pro-oxidative pathways of Hb-mediated lipid oxidation. Modified after Richards^6^.

Unless stated differently, wr-RBCs were stored at 3(± 1) °C after resuspension in 9 volumes of the solution of interest (see experiments i–v below in Fig. 2). Each experiment was repeated two times (n = 2), each time using a new batch of blood pooled from three to five fish. Within each experiment, all sample types were stored in duplicates (r = 2) and at each storage time point, one analysis of hemolysis was performed per sample (a = 1). Sampling was carried out for up to 16 days of storage at either 3(± 1) °C (experiment i, ii, iii and iv) or 9 (± 1) °C (experiment ii and v). In studies where RBCs were incubated in plasma, the RBCs had first undergone a washing procedure where after they were resuspended in trout blood plasma.

(i) To study the role of bacterial growth for hemolysis, RBCs were resuspended in 0.9% NaCl (i.e. saline) or blood plasma, and samples were prepared under sterile or non-sterile working conditions.
(ii) In the study of energy status, RBCs were resuspended in 3.8 mM glucose (Sigma-Aldrich; Stockholm, Sweden), a level measured in trout plasma, as well as in 7.6 mM lactate (Sigma-Aldrich) or 7.6 mM pyruvate (Sigma-Aldrich), the latter levels were selected as lactate/pyruvate formation from glucose is in a molar ratio of 2:1 during glycolysis. All three compounds were dissolved in saline, PBS pH 6.8 or PBS pH 8.0, and stored at 3(± 1) °C. Glucose consumption was measured in wr-RBCs resuspended in 3 volumes of blood plasma; stored at 9(± 1) °C.
(iii) For studies of hemolysis as a function of RBC membrane lipid oxidation at different pH´s, RBCs were resuspended PBS solution (pH 6.4, 6.8, 7.2, 7.6 or 8.0) prior to storage.
(iv) For deeper studies of the relation between RBC membrane lipid oxidation and hemolysis, RBCs were resuspended in PBS (pH 6.8) containing 200 µM D-(–) isoascorbic acid (AsA) (Sigma-Aldrich) without or with either (i) 0.5 mM or 1 mM hemin (Sigma-Aldrich) or (ii) 1 mM H_2_O_2_ (Sharlau) with 1 mM iron(II)sulfate heptahydrate (Sigma-Aldrich), prior to storage and sub-samples were taken up to 7 days of storage. In the oxidation studies (iv and v), measurement of hemolysis was conducted with 50 µL supernatant obtained as described below, while the remaining sample (1390 µL) was then stored in − 80 °C for subsequent thiobarbituric acid reactive substances (TBARS) analysis.
(v) To study the role of colloidal pressure (COP) on hemolysis, RBCs were resuspended in 3 volumes of plasma or PBS (pH 8.0) containing 3.8 mM glucose and a COP enhancing substance and stored at 9 (± 1) °C. To reach physiological COP (11.5 mmHg), 3.54 g/dL albumin (25% human serum albumin, Japan Blood Products Organization; Tokyo, Japan), 3.90 g/dL dextrin (Tokyo Chemical Industry Co., Ltd.; Tokyo, Japan) or 1.60 g/dL HMW dextran (40,000 Da, Fujifilm Wako Pure Chemical corp.; Tokyo, Japan) was added. One sample was adjusted to COP 30.5 mmHg with 7.0 g/dL albumin to investigate the effect of higher COP on hemolysis.

### Quantification of hemolysis

Hemolysis as a function of storage was quantified by analyzing Hb in supernatants obtained after centrifugation at 700×*g* as described in Ghirmai, Eriksson et al.^15^. In sub-study (iv), Hb of supernatants was also quantified spectrophotometrically with the Drabkin’s cyanometHb method and in sub-study (v) a kit was used to analyze total Hb concentration (Hemoglobin-B-test Wako,Fujifilm Wako Pure Chemical Corp., Tokyo, Japan).

### Microbiological analysis

An aliquot of 100 µL from the stored resuspended RBCs in sub-study (i) were spread on a plate count agar and if needed, ten-fold serial dilutions of the sample to 10^–2^, 10^–4^ and 10^–6^ with saline were performed; from which 100 µL was spread onto a plate count agar (PCA) (Sigma-Aldrich). The plates were incubated at 3(± 1) °C for 7 days whereafter colony forming units per mL (CFU/mL) were documented.

### Quantification of glucose concentration

An UV-based method with hexokinase (HK) and glucose-6-phosphate dehydrogenase (G6PD) was used for glucose measurement during storage of wr-RBCs in experiment (ii)^17^.

### Estimation of lipid oxidation in wr-RBCs

In experiments (iii) and (iv), total lipids were extracted from thawed resuspended RBCs according to a modified protocol of Lee, Trevino et al.^18^. Sample extraction was performed by vortexing with one volume chloroform:methanol (1:1) containing 0.05% w/v butylhydroxytoluene (BHT). Centrifugation at 2000×*g* for 6 min (4 °C) rendered phase separation. TBARS was analyzed in the water–methanol phase according to Schmedes and Hølmer^19^ using a spectrophotometer (Cary 60-UV–Vis, Agilent technologies, USA). The TBARS method is a commonly used lipid oxidation marker which measures free carbonyls such as malondialdehyde (MDA), formed when lipid hydroperoxides break down. Upon addition of thiobarbituric acid (TBA) and heating, a red colored carbonyl-TBA adduct is formed that can be measured at 532 nm. MDA was then used as an external standard to quantify the level of TBA-reactive substances (TBARS).

### Colloidal osmotic pressure

In experiment (v), a colloid osmometer (Osmomat 050, Gonotec, Berlin, Germany) with a 20 kDa cut-off membrane was used to measure COP. COP of rainbow trout (*Oncorhynchus mykiss*) blood plasma was estimated from measurements of blood plasma from five individual fish blood batches (n = 5) with two replicates of each analytical measurement (a = 2).

### Expression of results and statistical evaluations

Results are expressed as mean ± standard deviation (SD) based on the two replicate experiments (n = 2). An average of the two technical replicates (r) was used to make these calculations. One-way analysis of variance (ANOVA) was conducted to determine statistical significance between different RBC storage solutions and Tukey’s post-hoc test was conducted for a pairwise comparison when a significant effect was found, with a threshold of p < 0.05. All analyses were performed in R-studio version 3.5.1^20^.

## Results and discussion

### The effect of bacterial growth on hemolysis

**Figure 3:**
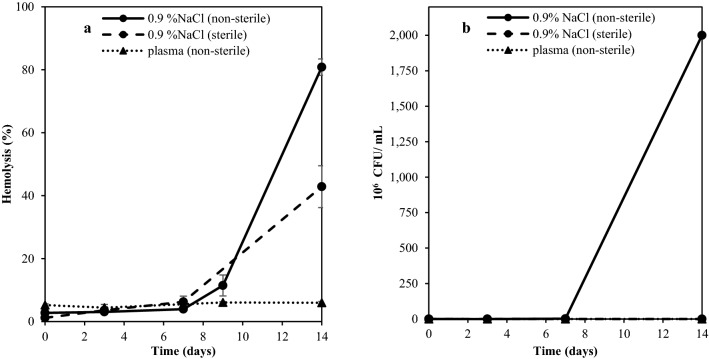
The relationship between hemolysis and microbial growth. (**a**) Hemolysis of wr-RBCs in saline or plasma as affected by sterile vs non-sterile storage conditions. (**b**) Total colony forming units (CFU) of sterile and non-sterile samples (expressed as 10^6^ CFU/mL) for sterile and non-sterile samples when stored at 3(± 1) °C.

In previous study, Ghirmai, Eriksson et al.^15^, it was difficult to conclude to what extent microbial contamination of wr-RBC suspensions affected storage-induced hemolysis. In the present study, aseptic techniques were applied to half of the samples, to compare hemolysis in sterile and non-sterile samples during cold storage. Figure 3a displays a modest increase of the RBC stability under aseptic conditions in saline, with significantly lower hemolysis in the sterile samples at day 14. Samples conducted under aseptic conditions showed no microbial growth (Fig. 3b), whereas the microbial count in non-sterile RBCs in saline had a lag phase of 7 days and reached up to 2 × 10^8^ CFU/mL at day 14. The threshold for bacterial levels during refrigerated storage of meat and fish products is 10^7^ to 10^9^ CFU/mL^21,22^. Surprisingly, RBCs stored in blood plasma which were processed under non-aseptic conditions also showed low microbial growth (Fig. 3b), with 0–10 CFU/mL up to 14 days of storage at 3(± 1) °C. This implicates that blood plasma contains antimicrobial agents that suppress microbial growth.

Blood cells such as leukocytes show rapid immunogenic responses to infections. Leukocytes generate the microbicidal agent hypochlorous acid; used as a commercial and household antimicrobial^23,24^. Several cationic proteins and peptides found in blood of eukaryotic organisms have also been identified with antimicrobial activity. For example, blood is described to exert antimicrobial properties through a mechanism that is peptide-based and oxygen-independent, by mobilizing cytotoxic proteins and peptides to the site of infection^23^. The rapid increase in bacterial growth of non-sterile samples at day 7 (Fig. 3b) could potentially be explained by the release of Hb as iron is an essential micronutrient for bacteria to proliferate^25^. Thus, lysis might have stimulated bacteria, rather than vice versa, meaning that accelerated bacterial spoilage of fish is an important aspect to consider when diluting the blood plasma in the process of rinsing fish fillets from blood. Our finding is partly contradictory to earlier findings that bleeding of fish reduces subsequent bacterial growth^14^, but at the same time clarifies that it may be the Hb that is critical for bacterial growth, rather than the plasma-derived nutrients such as glucose, vitamins and minerals alone. Indeed, the type of bacterial contamination occurring during fish handling at a primary processor vs. during sample preparation in a lab may also differ.

### Effect of energy status to samples on hemolysis

**Figure 4:**
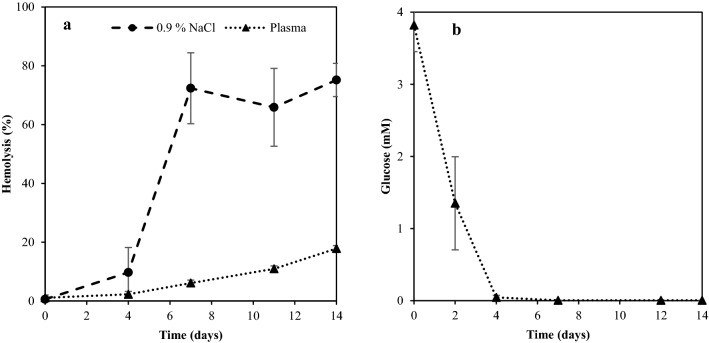
Comparison of hemolysis of wr-RBCs stored in in plasma versus in saline (**a**), and glucose consumption of RBCs incubated in plasma (**b**). Both a and b are conducted at 9(± 1) °C and the data represent mean ± SD (n = 2).

It was earlier found that saline fortified with glucose at a level endogenous to trout blood (6 mM) enhanced the lifespan of trout wr*-*RBCs compared to saline alone Ghirmai, Eriksson et al.^15^. To better understand how glucose affects RBC stability, hemolysis was related to the glucose consumption occurring during storage of wr-RBCs in plasma at 9 °C (Fig. 4). Unexpectedly, there was no clear relation between plasma glucose and hemolysis. Despite full consumption of glucose within 4 days of storage, the rate of hemolysis only slightly increased from day 4, resulting in less than 20% hemolysis by the end of the 14 days storage trial. The very high stability of wr-RBCs in plasma was in agreement with our earlier findings^15^, and the current observations indicate that plasma carry other RBC-stabilizing compounds, beyond glucose.

As trout RBCs contain mitochondria it enables aerobic metabolism, thus trout RBCs are not dependent on glucose alone as an energy source; instead, they can utilize pyruvate and lactate as well trough the Krebs cycle^26,27^. It has been suggested that pyruvate is a better substrate for aerobic metabolism as it produces more CO_2_ compared to glucose^27^. Increased metabolic activity was noticed in exhausted trout RBCs when lactate was added compared to glucose^27^. Ferguson and Storey^26^ revealed that reduction in the ATP-level yields loss in intracellular K^+^ and accumulation of Na^+^, as a consequence of K^+^/Na^+^ pump malfunction. Lactate or pyruvate at 7.6 mM vs glucose at 3.8 mM were therefore tested for their effect on wr-RBC stability during storage in saline as well as PBS at pH 6.8 and 8.0. All samples incubated in pH 8.0 had a prolonged lag-phase, with less than 20% hemolysis up to 12.4–18.4 days compared to 7.5–9.5 days for samples incubated in 0.9% NaCl or PBS pH 6.8. A significant increase in stability was found at day 16 for the glucose-fortified RBCs incubated at pH 8.0, compared to all RBC samples incubated in 0.9% NaCl and PBS pH 6.8 (Supplementary data).

This could be explained by increased glycolysis, as trout hexokinase (HK), the first enzyme in the glycolysis, have an optimal activity at pH 8.0 and phosphofructokinase (PFK) at pH 7.7^28^. An increase in glycolysis would result in increased production of the electron-energy rich molecule nicotinamide adenine dinucleotide (NADH), which is an important molecule for the metHb-reducing enzyme NADH-cytochrome b_5_ reductase. Further, it has been reported that human glucose-6-phosphate-dehydrogenase (G6PD) has an optimal activity at pH 8.0^29^. G6PD control the hexose monophosphate shunt (HMP) in which the second electron-energy rich molecule nicotinamide adenine dinucleotide phosphate (NADPH) is formed. This molecule is essential for the metHb reducing enzyme NADPH-flavin reductase^28^.

Glutathione (GSH) can detoxify the cell from reactive oxygen species (ROS) such as hydrogen peroxide through glutathione peroxidase (GPx). However, in the detoxification, oxidized glutathione (GSSG) is formed and for the regeneration of GSH glutathione reductase (GR) utilize NADPH. Thus, an increase in pH controls several pathways that could be of importance to control oxidation. The absense of increased RBC stability with pyruvate or lactate added, as well as the increased stability with added glucose at higher pH suggest that the production of NADH and NADPH might be critical for hemolysis as they control both metHb reducing enzymes and oxygen scavenging activities.

### The effect of pH on RBC membrane oxidation and hemolysis

**Figure 5:**
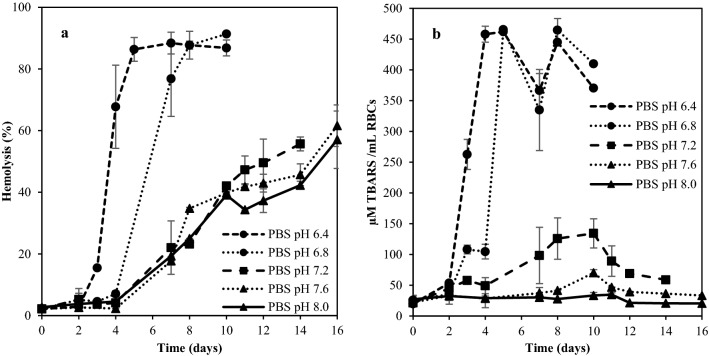
Effect of pH on hemolysis of wr-RBCs stored in 10 mM PBS (pH 6.4, 6.8, 7.2, 7.6 or 8.0) at 3(± 1) °C (**a**), and the effect of pH on TBARS formation (**b**). The results are visualized as mean ± SD, (n = 2).

The high PUFA content of RBC membranes, in combination with high concentration of oxygen and Hb make RBCs highly susceptible to lipid oxidation^30^. It has been reported that oxidative stress can cause formation of holes on the RBC membrane, leading to hemolysis^31,32^. To reveal reasons for the sudden lysis of wr-RBCs stored in saline (Figs. 3, 4), the relationship between RBC membrane lipid oxidation and hemolysis was therefore explored; and also how this relation is affected by pH. RBC samples were incubated in PBS with pH-values ranging from 6.4 to 8.0. This range was based on the pH of fresh trout blood plasma (7.8–8.0) and post-mortem cod and mackerel muscle pH (6.2–7.0)^33,34^. Rapid hemolysis and accumulation of the lipid oxidation marker TBARSs were observed for wr-RBCs stored at pH 6.4 and 6.8 (Fig. 5), indicating that at low pH, hemolysis might be driven by lipid oxidation of the RBC membrane. At pH 6.4, the hemolysis started already at day 2 and reached more than 50% hemolysis within 4 days of cold storage; with a similar trend in TBARS accumulation. RBCs stored at pH 6.8, 7.2, 7.6 and 8.0 were better preserved, and for all these samples, hemolysis started after day 4 of incubation. The hemolysis rate decreased with increasing pH; with 50% hemolysis being reached at day 5 (pH 6.8), 11 (pH 7.2), 14 (pH 7.6), and 15 (pH 8.0). TBARS followed a similar trend, with lower formation rate at increasing pH´s. At pH 7.6 and 8, TBARS were almost completely absent, indicating that other mechanisms were driving hemolysis, such as energy depletion as discussed above.

Due to the Root effect, small reductions in pH greatly reduce the oxygen saturation of teleost fish Hb; consequently, causing increased autoxidation^35,36^. In metHb, the porphyrin ring is loosely anchored to the globin and hemin is therefore readily released. Hemin is up to 60-fold less anchored to the globin in met-form^10^. Hemin (3+) and hemin (4+) can both react with lipid hydroperoxides (LOOH) and produce alkoxyl radicals (LO^**·**^) and peroxyl radicals (LOO^**·**^)^6^ which could destroy the RBC membrane. The observed difference in hemolysis and formation of lipid oxidation products between pH 6.4–7.2 and 7.6–8.0 (Fig. 5) might therefore indicate differences in hemin loss. This route could be further enhanced by the previously mentioned need of the electron carriers NADH and NADPH for the metHb reducing enzymes in RBCs, and the fact that these electron carriers are better generated at higher pH.

### Oxidation of RBC membrane lipids induced by hemin and H_2_O_2_ and its link to hemolysis

**Figure 6:**
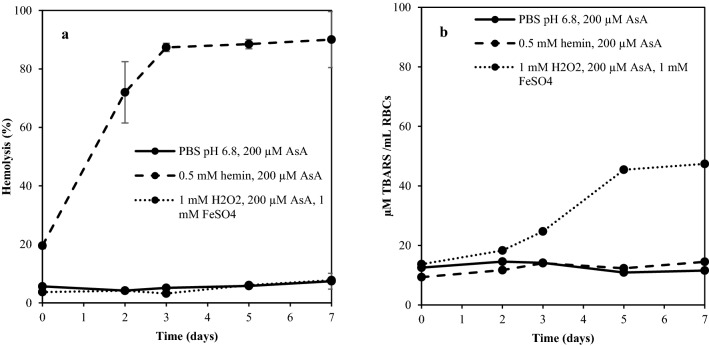
Relation between hemolysis (**a**) and RBC membrane lipid oxidation (**b**). RBCs were resuspended in PBS (pH 6.8) containing 200 μM ascorbic acid without or with either (i) 0.5 mM hemin or (ii) 1 mM hydrogen peroxide with 1 mM iron(II)sulfate, prior to storage at 3(± 1) °C. The results are visualized as mean ± SD, (n = 2).

Observing the necessity to control pH as a measure to limit RBC membrane lipid oxidation (Fig. 5), a study was designed to deeper evaluate the relationship of RBC membrane oxidation with hemolysis. Washed RBCs were resuspended in PBS (pH 6.8) with either hemin plus AsA, or H_2_O_2_ plus AsA and FeSO_4_ added to induce oxidation. Figure 6 visualize that the hemin-system surprisingly gave low TBARS formation but rapid hemolysis, indicating that accumulation of intracellular hemin driven by metHb formation could be a main or contributing cause for extensive hemolysis at pH < 7.2, not driven by membrane oxidation (Fig. 5). Studies on human RBCs have shown that hemin can induce hemolysis through aiding dissociation of membrane skeletal proteins^37^, i.e., a mechanism not linked to lipid oxidation. This finding implies that our observed rapid hemolysis pH < 7.2 could be driven by two different pathways. It can also not be ruled out that the AsA added to regenerate hemin (3+) from hemin (4+), exerted a separate antioxidative effect related to its radical scavenging properties^38^. This was supported by the lack of oxidation and hemolysis in the AsA-fortified control at pH 6.8.

The possibility that oxidation and hemolysis can develop independently was supported by the fact that RBC membrane lipid oxidation increased after addition of H_2_O_2_/Fe/AsA without stimulating hemolysis (Fig. 6). In presence of activated oxygen species, ferrous iron can catalyze hydroxyl radical formation (**·**OH) from H_2_O_2_ through the Fenton reaction^39,40^. Hydroxyl radicals (**·**OH) are very strong oxidants, which initiate lipid oxidation by abstracting a hydrogen atom directly from PUFAs, or from aqueous molecules as proteins closer to the **·**OH generation site, which then transfer the oxidative attack to the PUFAs^41^. At post-mortem pH’s typical for fish, i.e., around 6.2–7.0, the neutral superoxide radicals (HO_2_**·**) are found in substantial concentrations, causing fast generation of H_2_O_2_ with its conjugate base, superoxide anion radicals (**·**O_2_^−^), which are formed in the autoxidation of oxyHb^6^. In addition to be a substrate in hydroxyl radical formation, H_2_O_2_ can convert oxyHb to metHb^42^ and can also oxidize deoxyHb to ferrylHb (ferrylHb(4 +) = O), which in turn may react with another deoxyHb to produce metHb. MetHb can then further react with H_2_O_2_ and form ferrylHb radicals (ferrylHb^(**·**+)^(4 +) = O). The ferrylHb radical is a direct initiator of lipid oxidation and can also propagate lipid oxidation^6^. Altogether, to completely understand the role of fish RBC membrane lipid oxidation in hemolysis, deeper evaluations of different oxidation mechanisms are needed combined with monitoring of a broader spectra of oxidation products, e.g., free radicals.

### Colloidal osmotic pressure and hemolysis

Albumin has previously been found to have a stabilizing effect on wr-RBCs from trout^15^. This effect was hypothesized to be due to colloidal osmotic pressure (COP). In the present study, the contribution of COP to the high stability of trout wr-RBCs in plasma was investigated by mimicking the trout plasma COP with albumin, dextrin, or high molecular weight (HMW) dextran. Our hypothesis was that if a RBC stabilizing effects could be obtained with the chosen polysaccharides, it might both explain the protective role of plasma, and provide an opportunity to integrate new RBC stabilizing strategies into the processes of the fish industry. Currently, both albumin and HMW-dextran are used as plasma replacement fluids^43,44^. Dextrin was chosen for its similarities to the plasma replacement fluid hydroxyethyl starch (HES), a starch derivative that is chemically modified to have reduced susceptibility to non-specific hydrolysis by amylase in the blood^45^.

**Figure 7:**
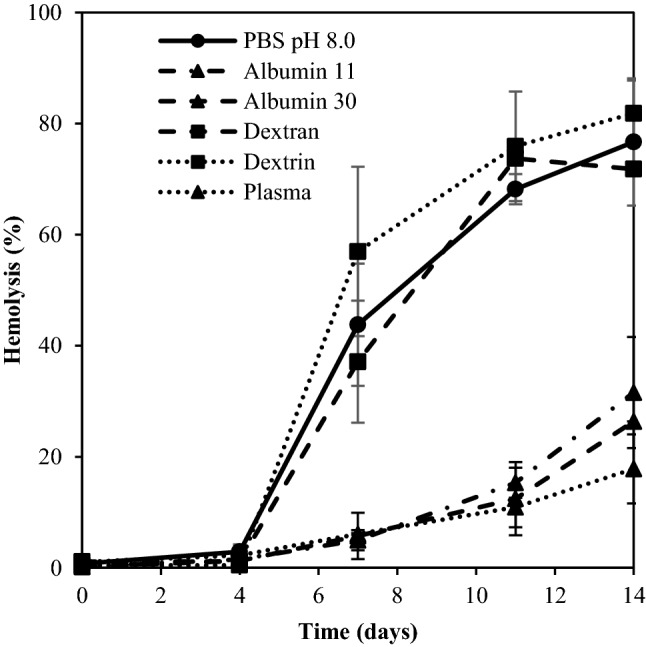
Effect of colloidal osmotic pressure (COP) from plasma, albumin (11 and 30 mmHg), dextrin, or HMW dextran, when stored at 9(± 1) °C. The data represent mean ± SD, (n = 2).

In the studies of COP (Fig. 7), only wr-RBC suspensions containing albumin significantly delayed hemolysis in a similar manner as blood plasma, i.e., up to day 14. Albumin is a negatively charged protein that could potentionally attach to the RBC membrane and bind sodium ions; protecting the RBC from accumulating water^46^. It is also possible that albumin did not act by its effect on the COP, but by scavenging free radicals, and thereby preventing hemolysis induced by oxidation^47^. Albumin has, together with the Hb-binding haptoglobin and heme-binding hemopexin, been ascribed an important role in the defense mechanisms toward free heme in the blood^25,48^.

## Conclusions

This study revealed that a combination of several features related to blood and post mortem muscle affect trout RBC stability, some which may explain the highly protective role observed for blood plasma. In absence of plasma, pH-values < 7.2 strongly triggered hemolysis with a parallel increase in RBC membrane lipid oxidation. A potential link between the two events was however put in doubt as hemolysis also was initiated by free hemin, without the formation of secondary lipid oxidation products. Thus, hemin-release triggered by metHb formation could be a direct route to lysis below pH 7.2. At higher pH, 7.6–8.0, the hemolysis rate seemed to be driven primarily by other, yet unknown, factors. Bacterial growth was excluded as a main reason for hemolysis of RBCs, whereas the presence of glucose in combination with high pH significantly stabilized the RBCs. Storing the RBCs at pH 8 in presence of 3.8 mM glucose and albumin, at or above a level providing physiological COP, resulted in very low hemolysis; similar to that of RBCs incubated in blood plasma. A role of the COP per se could however not be proven, pointing at specific roles of albumin linked e.g. to radical scavenging or ion binding.

Together these findings can gradally pave the way for new strategies to control hemolysis in fish, although they first must be confirmed in fish muscle-based systems. Among tentative new strategies, which via a higher RBC stability, could prevent Hb-mediated lipid oxidation would be to rinse or submerge fish fillets in solutions isotonic to the fish muscle, and with pH above neutrality. These solutions could also be enriched with glucose and/or albumin. Indeed, such strategies could also be highly suitable for the more challenging blood-rich filleting side streams, allowing for more responsible use of our caight or harvested fish for food production, rather than to feed.

## Supplementary Information

Supplementary Information.
