# Supplementary material for: Exploring how plasma- and muscle-related parameters affect trout hemolysis as a route to prevent hemoglobin-mediated lipid oxidation of fish muscle
Source: Sci Rep. 2022 Aug 4;12:13446. doi: 10.1038/s41598-022-16363-4 (PMC9352706; doi:10.1038/s41598-022-16363-4)
Supplement: Supplementary file 1 — Supplementary Information. [file 41598_2022_16363_MOESM1_ESM.docx]

**Title:** Exploring how plasma- and muscle-related parameters affect trout hemolysis as a route to prevent hemoglobin-mediated lipid oxidation of fish muscle

Ghrimai, S.*^a^,Wu, H.^a^, Axelsson, M.^b^, Matsuhira, T.^c^, Sakai, H.^c^ and Undeland, I.^a^

**Author names:**

*Corresponding author

*Semhar Ghirmai*^a^*

Email address: semhar.ghirmai@chalmers.se

Tel. no.: +4631-772 68 63

*Haizhou Wu^a^*

Email address: [haizhou@chalmers.se](mailto:haizhou@chalmers.se)

*Michael Axelsson^b^*

Email address: [Michael.axelsson@bioenv.gu.se](mailto:Michael.axelsson@bioenv.gu.se)

*Takashi Matsuhira^c^*

Email address: [mattu@naramed-u.ac.jp](mailto:mattu@naramed-u.ac.jp)

*Hiromi Sakai^c^*

Email address: [hirosakai@naramed-u.ac.jp](mailto:hirosakai@naramed-u.ac.jp)

*Ingrid Undeland^a^*

Email address: [undeland@chalmers.se](mailto:undeland@chalmers.se)

Tel. no.: + 4631-772 38 20

**Affiliations:**

^a^Chalmers University of Technology, Department of Biology and Biological Engineering, Division of Food and Nutrition Science, SE-412 96 Gothenburg, Sweden

^b^Gothenburg University, Department of Biological and Environmental Sciences, Medicinaregatan 18a, SE-413 90 Gothenburg, Sweden

^c^Department of Chemistry, Nara Medical University, 840 Shijo-cho, Kashihara, Nara 634-8521, Japan

**Supplementary Data**


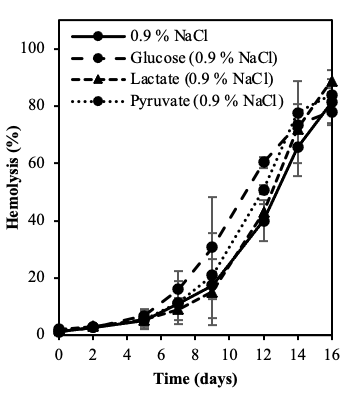

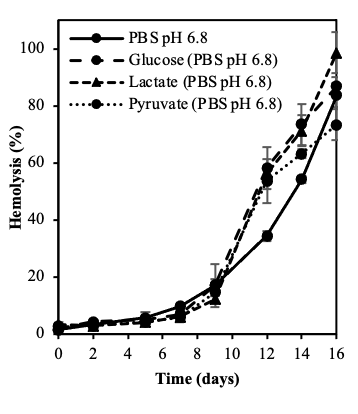

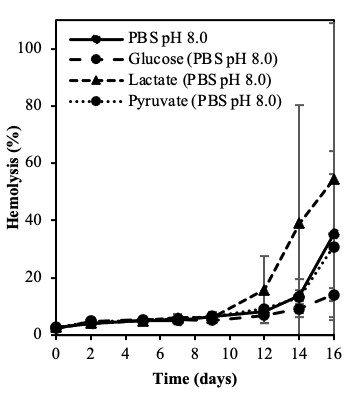


**Fig. 1.** Development of hemolysis in glucose, lactate or pyruvate enriched solutions based on 0.9% NaCl (panel a), PBS pH 6.8 (panel b) or PBS pH 8.0 (panel c).

**a**

**b**

**c**
